# Development of a spontaneous preterm birth predictive model using a panel of serum protein biomarkers for early pregnant women: A nested case–control study

**DOI:** 10.1002/ijgo.15876

**Published:** 2024-08-27

**Authors:** Shuang Liang, Yuling Chen, Tingting Jia, Ying Chang, Wen Li, Yongjun Piao, Xu Chen

**Affiliations:** ^1^ Tianjin Central Hospital of Gynecology Obstetrics/Nankai University Affiliated Maternity Hospital Tianjin China; ^2^ Tianjin Key Laboratory of Human Development and Reproductive Regulation Tianjin China; ^3^ School of Life Sciences Tsinghua University Beijing China; ^4^ School of Medicine Nankai University Tianjin China

**Keywords:** biomarker, first trimester, liquid chromatography tandem mass spectrometry, machine learning, prediction model, preterm birth, soluble fms‐like tyrosine kinase‐1

## Abstract

**Objective:**

To develop a model based on maternal serum liquid chromatography tandem mass spectrometry (LC–MS/MS) proteins to predict spontaneous preterm birth (sPTB).

**Methods:**

This nested case–control study used the data from a cohort of 2053 women in China from July 1, 2018, to January 31, 2019. In total, 110 singleton pregnancies at 11–13^+6^ weeks of pregnancy were used for model development and internal validation. A total of 72 pregnancies at 20–32 weeks from an additional cohort of 2167 women were used to evaluate the scalability of the model. Maternal serum samples were analyzed by LC–MS/MS, and a predictive model was developed using machine learning algorithms.

**Results:**

A novel predictive panel with four proteins, including soluble fms‐like tyrosine kinase‐1, matrix metalloproteinase 8, ceruloplasmin, and sex‐hormone‐binding globulin, was developed. The optimal model of logistic regression had an AUC of 0.934, with additional prediction of sPTB in second and third trimester (AUC = 0.868).

**Conclusion:**

First‐trimester modeling based on maternal serum LC–MS/MS identifies pregnant women at risk of sPTB, which may provide utility in identifying women at risk at an early stage of pregnancy before clinical presentation to allow for earlier intervention.

## Synopsis

A novel sPTB predictive panel with four proteins, including sFlt‐1, MMP‐8, ceruloplasmin, and SHBG, at 11–13^+6^ weeks of pregnancy was developed.

## 1 INTRODUCTION

Preterm birth (PTB) is defined as delivery before 37 weeks of pregnancy, and it is a major pregnancy issue that affects children.^1^ According to WHO, PTB is the leading cause of perinatal morbidity and mortality globally.^2^ Moreover, the survivors among these premature neonates are at an increased risk of long‐term morbidities, such as cerebral palsy, neurodevelopmental impairment, schizophrenia, and anxiety disorder,^3^, ^4^, ^5^ that result in a large economic burden.^6^, ^7^ The application of interventions to prevent PTB remains challenging because of the inability to assess the true risk of preterm labor. Great effort has been made to predict PTB; however, known screening methods or risk factors, such as cervicovaginal fetal fibronectin testing (a biochemical marker of cervical changes that may subsequently result in labor and delivery), history of spontaneous preterm birth (sPTB), and short cervical length, have not improved outcomes of PTB in clinical practice.^8^, ^9^, ^10^

The predictors of sPTB need to be further determined. According to the latest studies in molecular biology, sPTB is a heterogeneous disorder with multiple etiologies, including intrauterine infection/inflammation, placental protein/hormonal disorders, matrix remodeling, and abnormal allogenic recognition.^11^, ^12^, ^13^ This heterogeneity suggests that it cannot be accurately predicted by a single indicator. The widespread use of liquid chromatography–tandem mass spectrometry (LC–MS/MS), which is an advanced analytical technique used for the separation, identification, and quantification of compounds in a sample, enables the detection of multiple protein biomarkers simultaneously, providing new hope for clinical prediction of the disorder. Moreover, models built from proteomic (large‐scale study of proteins, analyzing proteins to identify potential biomarkers for disease) data predict sPTB with higher accuracy and earlier in pregnancy than other omics models. In a Proteomic Assessment of Preterm Risk (PAPR) project, a research initiative focused on using proteomic analysis to assess the risk of preterm birth, Saade et al.^7^ used MS to assess maternal circulating proteomic profiles in the second trimester and identified two proteins related to PTB: insulin‐like growth factor‐binding protein 4 and sex‐hormone‐binding globulin (SHBG); this provides feasibility for detecting predictive indicators of sPTB by MS.

Preterm birth has been recently reported to be a primary placental disorder, which is the result of angiogenesis disorders typified by shallow trophoblast invasion and deficient spiral artery conversion in early pregnancy.^14^, ^15^ Kim et al.^16^ observed that patients delivered preterm had a greater degree of failure of transformation of the spiral arteries in the myometrial and decidual segments than women who delivered at term. Most of the current research on the prediction of PTB is based on biomarkers of second and late trimester, therefore, the fundamental role of placental dysfunction in early trimesters in PTB cannot be reflected, whereas other ‘placenta‐originated disease’, such as pre‐eclampsia, was predicted by maternal serum in early pregnancy and the model performed well. Identification of early biomarkers is necessary to develop treatment strategies that reduce the impact of prematurity.

Here, we performed a proteomic profile with LC–MS/MS at 11–13^+6^ weeks of pregnancy, based on 44 selected candidate biomarkers found to be involved in the pathologic processes of sPTB and developed a predictive model with four proteins, including soluble fms‐like tyrosine kinase‐1 (sFlt‐1), matrix metalloproteinase 8 (MMP‐8), ceruloplasmin, and SHBG, which had a distinguishing performance. Then, an additional evaluation was proceeded in women at 20–32 weeks of pregnancy to evaluate the scalability of the model across different gestational weeks. This study provides a new panel and predictive model for sPTB and provides the potential to improve treatment and prognosis for clinical practice.

## 2 MATERIALS AND METHODS

### 2.1 Ethics

This nested case–control study was approved by the Ethics Committee of Tianjin Central Hospital of Obstetrics and Gynecology (2020KY023, April 2, 2020).

### 2.2 Participants

**FIGURE 1:**
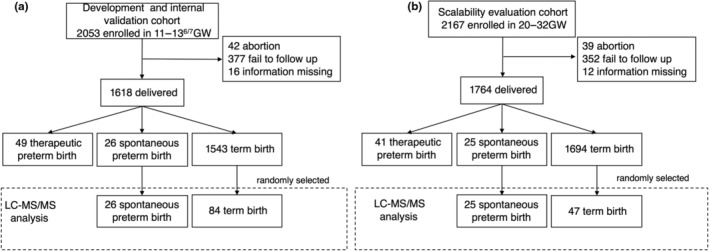
Workflow of participant enrollment for the (a) development and (b) scalability evaluation phases. LC–MS/MS, liquid chromatography tandem mass spectrometry.

A previous development nested case–control study was conducted in a cohort of 2053 women enrolled between January 1, 2018, and January 31, 2019, at Tianjin Central Hospital of Obstetrics and Gynecology. Women were included in this study after written consent was obtained from them. Healthy pregnant women between the ages of 20 and 40 years who presented to Tianjin Central Hospital of Obstetrics and Gynecology for delivery were eligible. The development cohort was based on singleton pregnancies at the time of prenatal aneuploidy serum screening at 11–13^+6^ weeks of pregnancy. An additional cohort of 2167 women were used to evaluate the scalability of the prediction model. The sample size of the case and control groups was calculated in a ratio of 1:3 in the development cohort and 1:2 in the scalability cohort, respectively. Finally, LC–MS/MS analysis was performed on the sPTB and matched control groups in both the development and validation cohorts (Figure 1). Gestational age was determined based on the first day of the last menstrual period and was corroborated by ultrasound dating. Maternal sociodemographic data were recorded by trained healthcare staff. Body mass index (calculated as weight in kilograms divided by the square of height in meters) was derived from the patient's height and self‐reported prepregnancy weight. Following delivery, data were collected for maternal and infant outcomes and complications. All deliveries were classified as term (≥37 weeks), sPTB (including preterm premature rupture of membranes), or medically indicated preterm births. All sPTB cases and controls in this study were individually adjudicated by the chief medical officer, and discrepancies were clarified with the principal investigator at the clinical site. With the exception of the director of clinical operations, all laboratory and data analysis personnel were blinded to the clinical data.

### 2.3 Sample collection

Maternal whole blood was placed in a 4°C refrigerator for no more than 2 h until centrifugation (2200*g* at 4°C for 10 min). After a 10‐min room temperature clotting period, 0.5‐mL serum aliquots were carefully separated and stored at −80°C until subsequent analysis.

### 2.4 Sample preparation

An extensive literature review was performed, and from this literature, 44 candidate biomarkers that have been found to be associated with the pathologic processes implicated in PTB were selected. In Table S1, the candidate biomarkers and molecular pathways are categorized along with the corresponding literature reviews that highlight the role of each biomarker in the pathologic processes implicated in PTB. The corresponding unique peptides used for target protein identification based on MS are listed in Table S2. All of these peptides were synthesized and purified to above 90% purity. For protein digestion of serum samples, 140 μL of 8 m urea in phosphate‐buffered saline buffer was added to 2 μL serum, followed by tris(2‐carboxyethyl)phosphine (TCEP) reduction and chloroacetamide alkylation. Then, trypsin was added at a 1:50 ratio (micrograms of enzyme to micrograms of protein). Digestion was performed at 37°C overnight. Peptides were acidified to a final concentration of 0.1% trifluoroacetic acid and desalted by tC18 Sep‐Pak columns (Waters). The elution was completely dried using a SpeedVac centrifuge at 45°C, and peptides were suspended in 100 mM triethylammonium bicarbonate (TEAB) buffer. The synthesized peptides were also dissolved in 100 mM TEAB buffer at an appropriate concentration, reduced by TCEP and alkylated by chloroacetamide. Synthesized peptides and serum peptides were labeled by TMT6plex reagents. Each Tandom Mass Tag (TMT) group contained TMT126‐labeled synthesized peptides and four serum samples labeled with TMT128–131 reagents. After quenching the labeling reaction by 5% hydroxylamine, different TMT‐labeled samples were mixed together and desalted by homemade C18 StageTips. The collection material was dried and suspended in 0.1% formic acid for MS analysis.

### 2.5 LC–MS/MS analysis

Details of LC–MS/MS profiling procedures are listed in Supplemental methods S1.

### 2.6 Outcomes and measurements

The outcome measures for this study were sPTB less than 37 weeks of pregnancy.

### 2.7 Data preprocessing and marker selection

Protein markers with more than 20% missing values were excluded from the analysis. The missing values of the remaining markers were imputed with mean values using the imputeTS R package. Then, machine learning models were used to select relevant protein markers for predicting PTB. This process can be seen as a feature selection problem in machine learning, aiming to determine a small subset of features (proteins) by eliminating irrelevant ones. Feature selection approaches can be broadly divided into two types: filters and wrappers. The filters employ independent metrics, that is correlation coefficients, to evaluate features and select the best subset based on a cut‐off. On the other hand, the wrappers use machine learning algorithms to determine the best subset. The filters are faster than wrappers, but the interaction with the final prediction model is ignored. Wrappers can identify the most discriminative subsets for the prediction model, though they are computationally expensive. Given that the dimensionality (the number of protein markers) of our data was not very high, we used the wrapper approach with sequential forward selection (SFS) to select relevant markers for sPTB prediction. Starting with the empty set, the selection model added one marker at a time and repeated the procedure until the best marker subset was obtained. A support vector machine (SVM) was used as an evaluation algorithm for candidate subsets generated from each round of iteration, and the five‐fold cross validation accuracy was used as a selection criterion. For example, with a data set consisting of n proteins denoted as *P*_1_, *P*_2_, …, *P*_*n*_, the process begins with an empty best set. We first constructed prediction models using SVM for each protein individually. The protein with the best prediction accuracy was added into the best set. Next, one of the remaining proteins not in the current best set was chosen, combined with the current best set, and prediction models were constructed. The protein forming the best combination (yielding the best prediction accuracy) with the current best set was then added to it. This process was repeated until no improvement in performance was observed.

### 2.8 PTB prediction model development and scalability evaluation

Logistic regression (LR), SVM, and random forest (RF) were used to construct predictive models. To perform the internal model validation, we used five‐fold cross validation. In the five‐fold cross validation, the data were randomly divided into five pieces. Four pieces were used for the training, and the remaining one was used for the testing. Model discrimination was assessed by accuracy, sensitivity, specificity, precision, F‐measure, and the area under the receiver operating characteristic curve (AUC), and the calibration was assessed by the calibration plot. To evaluated scalability of the model, an independent cohort consisting of different gestational weeks (20–32) were selected for LC/MS–MS analysis. Then, we also evaluated the model performances in terms of discrimination and calibration as was done in the development data set. A Scikit‐learn python machine learning package (v1.2.0) was used for model development and scalability evaluation.

## 3 RESULTS

### 3.1 Clinical characteristics

Of the 2053 participants in the development cohort, 42 (2.05%) were excluded because of abortion or termination of pregnancy, 16 participants (0.78%) provided insufficient information, and 377 (18.36%) were lost to follow up. Of the remaining 1618 participants, 75 of them had PTB (4.64%), including 26 (34.67%) spontaneous and 49 (65.33%) medically indicated PTB. Eighty‐four controls were randomly selected from 1543 participants with normal delivery. There were no statistically significant differences between the case and control groups in terms of maternal age, educational level, or sampling time (Table 1), with the exception that participants with sPTB were more likely to have had one or more previous PTB and more likely to have conceived the current pregnancy through assisted reproduction (*P* = 0.012). A total of 25 cases of sPTB and 47 controls from an additional cohort of 2167 participants were enrolled in the scalability evaluation phase. For three of the scalability cohort, samples were degraded, so there were only 47 controls in the scalability cohort. The characteristics of the PTB cases and controls selected for the overall scalability evaluation cohort were not significantly different. The characteristics of these participants are summarized in Table S3.

**TABLE 1:** Demographic characteristics of the participants.^a^

Characteristics	Preterm (*n* = 26)	Term (*n* = 84)	*P* value
Age, years	31.31 ± 0.53	30.14 ± 0.43	0.164
Prepregnancy BMI			0.180
<18.5	4 (15.38%)	15 (17.86%)	
18.5–24.9	19 (73.08%)	57 (67.86%)	
≥25	3 (11.54%)	12 (14.29%)	
Height, cm	162.58 ± 0.63	164.00 ± 0.59	0.104
Educational level			0.993
<College	3 (11.54%)	10 (11.90%)	
=College	21 (80.77%)	67 (79.76%)	
>College	2 (7.69%)	7 (8.33%)	
Cigarette smoking	1 (3.85%)	1 (1.19%)	‐
Multigravida	6 (23.08%)	21 (25.00%)	0.040
Preterm delivery history	3 (11.54%)	1 (1.19%)	1.000
Term delivery history	3 (11.54%)	20 (23.81%)	0.842
Primigravida	20 (76.92%)	63 (75.00%)	0.012
Assisted reproduction	3 (11.54%)	0 (0.00%)	0.535
Gestational days at serum collection	88.92 ± 0.76	88.35 ± 0.46	
Bleeding during pregnancy, before 12 weeks	8 (30.77%)	14 (16.67%)	0.116

Abbreviation: BMI, body mass index (calculated as weight in kilograms divided by the square of height in meters).

^a^ Data are presented as mean ± standard deviation or as number (percentage).

### 3.2 Model development and internal validation

**FIGURE 2:**
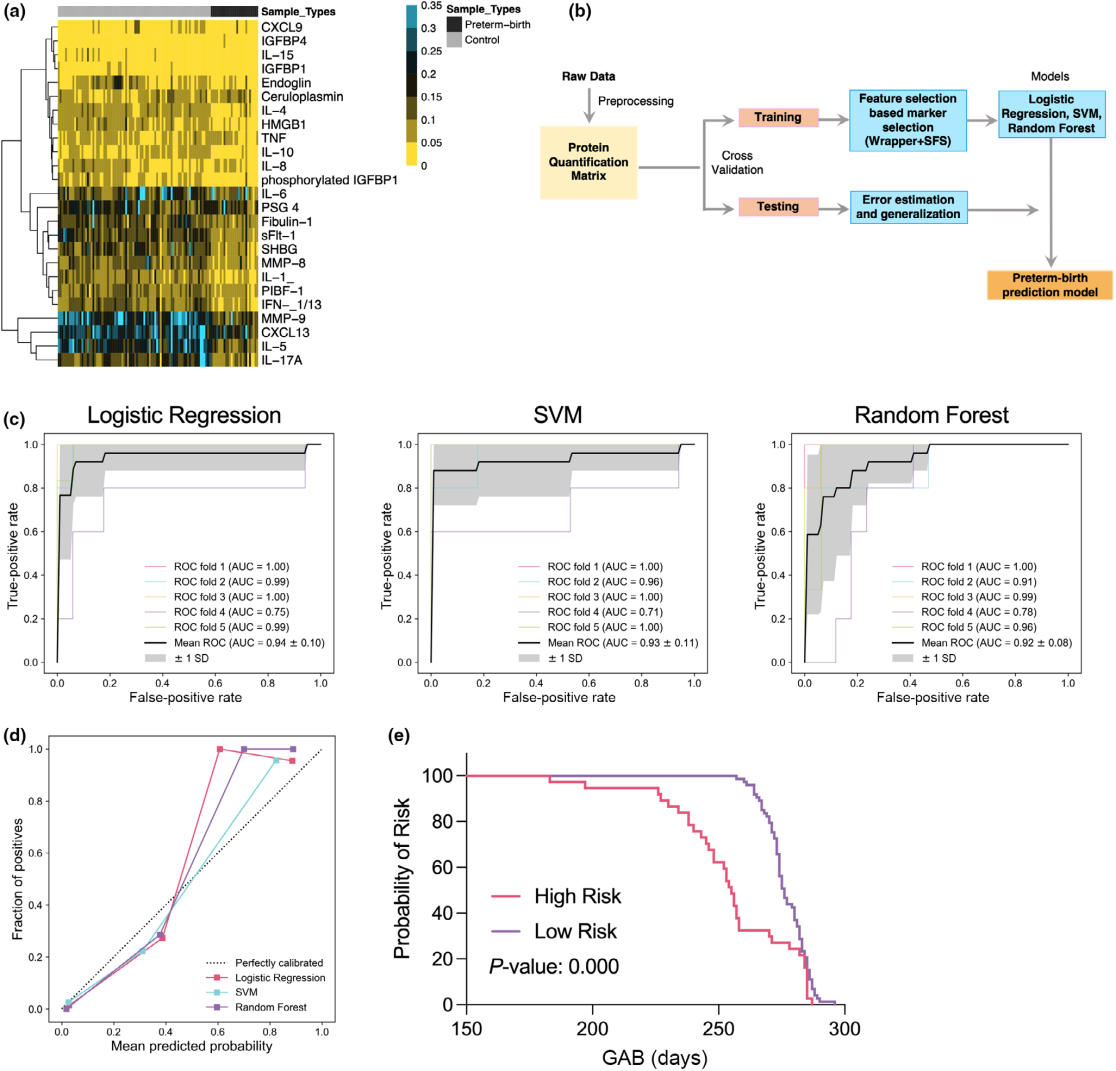
Spontaneous preterm birth (sPTB) prediction model development. (a) Unsupervised clustering analysis of protein markers between the sPTB and control groups in the training data set. (b) Workflow for the development and validation of the prediction model. (c) Receiver operating characteristics analysis of three different machine learning models five‐fold cross validation. (d) Calibration plot of three prediction models. (e) Kaplan–Meier analysis of high and low risk groups. AUC, area under the curve; GAB, gestation at birth; ROC receiver operating characteristics; SVM, support vector machine.

Of the 44 candidate biomarkers involved in the pathologic processes of sPTB, 35 were detected in the maternal serum samples by LC–MS/MS analysis. Ten of the 35 proteins detected had more than 20% missing values, so these 10 proteins were removed. Of the remaining proteins, two of them (interferon alpha‐13 and interleukin‐5) had missing values in eight samples, and these missing values were imputed with mean. After processing of missing values, 25 proteins were finally used for downstream analysis, and the heatmap of the data is shown in Figure 2a. The most discriminative markers sFlt‐1, MMP‐8, ceruloplasmin, and SHBG, which should be used in combination, were selected as final sPTB prediction markers based on the feature selection approach. Then, predictive models were constructed using machine learning algorithms based on those four markers (Figure 2b). LR were found to result in best performance with a classification accuracy of 0.92, followed by lower accuracy values with SVM (0.91) and RF (0.87) (Table 2). The average AUC of 0.94, 0.93, and 0.92 were achieved in the five‐fold cross validation for LR, SVM, and RF, respectively (Figure 2c). A model calibration plot is shown in Figure 2d. The participants were then divided into high‐risk or low‐risk groups according to a predictive probability cutoff which was twice the average sPTB rate in China (7.3% × 2 = 14.6%),^17^ and Kaplan–Meier analysis was then conducted between these two groups (Figure 2e). The high‐risk group delivered earlier than the low‐risk group (*P* = 0.000).

**TABLE 2:** Performance estimation of preterm birth prediction model in terms of classification accuracy, sensitivity, specificity, precision, and F‐measure on five‐fold cross validation.

Models	Acc	Sen	Spe	Precision	F‐measure
LR	0.92	0.77	0.97	0.89	0.81
SVM	0.91	0.88	0.92	0.79	0.82
RF	0.87	0.63	0.95	0.81	0.68

Abbreviations: Acc, accuracy; LR, logistic regression; RF, random forest; Sen, sensitivity; Spe, specificity; SVM, support vector machine.

### 3.3 Scalability of the model across different gestational weeks

**FIGURE 3:**
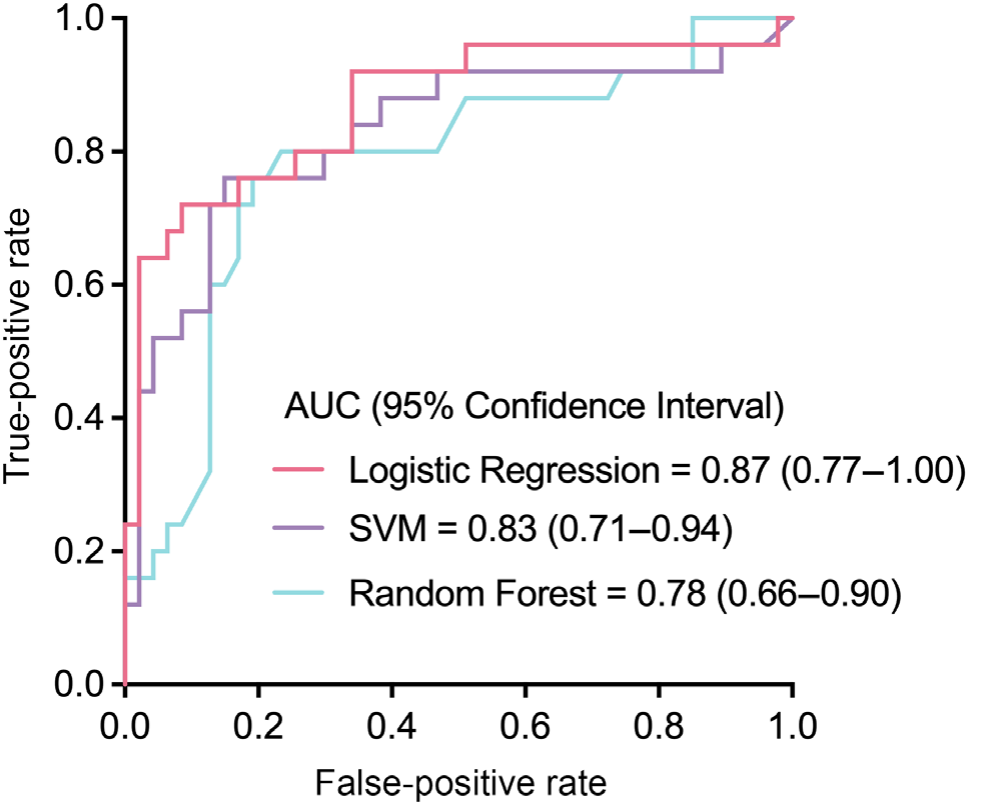
Receiver operating characteristics analysis of three prediction models on a scalability evaluation set. AUC, area under the curve; SVM, support vector machine.

To further evaluate whether the prediction model is expandable for prediction of sPTB in second and third trimesters, the developed model was evaluated on an independent cohort comprising different gestational weeks (20–32 weeks). In the scalability evaluation set, LR still achieved the best performance, followed by RF and SVM (Table S4). and receiver operating characteristics curves are shown in Figure 3. As shown in the figure, LR achieved the best performance with an AUC of 0.87 (95% confidence interval [CI] 0.77–1.00), followed by SVM (AUC 0.83, 95% CI 0.71–0.94) and RF (AUC 0.78, 95% CI 0.66–0.90), respectively. Model calibration plot is shown in Figure S1.

## 4 DISCUSSION

In this study, we developed a novel sPTB predictive panel with four proteins, including sFlt‐1, MMP‐8, ceruloplasmin, and SHBG, at 11–13^+6^ weeks of pregnancy from 44 molecules that were found to be associated with pathologic processes of PTB. This model trained with logistic regression could accurately distinguish sPTB from normal deliveries with an AUC of 0.94 on five‐fold cross validation, and it still worked well in an independent set of women at 20–32 weeks of pregnancy with an AUC of 0.868. These four biomarkers were first combined to predict sPTB, and the model showed good and stable performance. This early detection may have the potential to improve treatment and prognosis in clinical practice.

Previous studies have documented several indicators for the prediction of sPTB, but the prediction of sPTB still remains a challenge. A history of sPTB is considered to be the best measure of clinical risk to date^7^; however, more than 50% of PTB occur in low‐risk groups.^18^ Cervicovaginal fetal fibronectin can be a useful biomarker for predicting PTB within 7–14 days in women with contractions and mild acute preterm labor, but the predictive value of fetal fibronectin for PTB more than 14 days after testing is poor.^19^ It has been reported that using combinatorial biomarkers for PTB prediction had more predictive value than a single biomarker because of the heterogeneity of PTB pathology.^20^

Preterm birth has been reported to be rooted in aberrant placentation at the end of the first trimester. Classically, trophoblast cells invade the decidual and myometrial segments of the spiral arteries, and this physiologic transformation of the spiral arteries leads to substantial dilation of these vessels, which is considered key to accommodating the increased blood flow to the uteroplacental circulation.^21^, ^22^, ^23^ Accumulative observations have indicated that PTB is associated with failure of the physiologic transformation of the spiral arteries, which is considered canonical in the setting of pre‐eclampsia.^24^, ^25^, ^26^ The rate of failure of physiologic transformation of the myometrial segment of the spiral arteries was reported to be significantly higher for patients with PTB than for those who had a term delivery (30.9% vs 13.6%, *P* = 0.004).^17^ Our model based on the sampling window of 11–13^+6^ weeks of pregnancy, which coincided with the timing of routine obstetrical visits, enables early detection of women at risk of sPTB.

To our knowledge, this is the first time that sFlt‐1, which is related to abnormal placentation, has been used as one of the factors to predict sPTB. sFlt‐1 is an antiangiogenic substance secreted by trophoblasts, endothelial cells, and monocytes and has been proposed to be responsible for an antiangiogenic state and systemic endothelial dysfunction.^27^ The concept of “placental hypoperfusion” has recently been proposed and manifests as a series of pregnancy‐related complications caused by vascular remodeling disorders that can be predicted by sFlt‐1 in early pregnancy, including pre‐eclampsia and PTB.^11^, ^28^, ^29^, ^30^ Previous studies have reported that sFlt‐1 levels were higher in cases of PTB. Our study supports these findings; furthermore, our multivariate predictive model suggests that sFlt‐1 may have predictive value for this group of patients.

Intra‐amniotic infection/inflammation and placental hormonal disorders have been associated with PTB.^11^ Serum ceruloplasmin is secreted by the liver and is known to be an acute‐phase reactant that modulates inflammatory responses. It was reported to be related to premature rupture of the membranes and PTB. MMP‐8, also known as neutrophil collagenase, is secreted by a variety of inflammatory cells. MMP‐8 is present at the initial stages of the inflammatory process and has been reported to play a role in PTB. SHBG is secreted by the liver and placenta, and together with insulin‐like growth factor‐binding protein 4, has been reported to be a predictor of PTB, as SHBG controls androgen and estrogen actions in the placental‐fetal unit in response to upstream inflammatory signals.^7^ Therefore, our panel includes multiple mechanisms of PTB, which may be more valuable for predicting PTB, because of its heterogeneity.

Preterm birth is a multi‐mechanism disorder and our panel includes molecules involved in different mechanisms of PTB with stable performances, indicating that the prediction of PTB should be based on multiple perspectives. Early detection may have the potential to improve prognosis in clinical practice on the basis of additional surveillance and interventions (corticosteroids and magnesium sulfate), which have well‐established benefits for newborns. Large‐sample validation research in the future and would enable earlier prediction.

There are several strengths in the present study. First, we chose a multi‐index prediction model related to the three major causes of PTB (vascular remodeling disorders, intra‐amniotic infection, and placental hormonal disorders), which was in line with the heterogeneity of PTB. Second, we used early pregnancy as the modeling time window based on the placentation abnormalities occurring in the early stage. Early prediction may provide more time for future intervention, just as pre‐eclampsia prediction did. Third, we used an independent set of women at 20–32 weeks of pregnancy to evaluate its scalability, which showed that our model could be applicable for various periods of gestation.

There are still limitations in the present study. First, the study was a retrospective nested case–control study; therefore, mixed factors could not be well controlled. Second, the participants were exclusively from a single center and the data set was small, further optimization of the model requires large‐sample, multicenter validation. Third, this study was not stratified to assess these markers for sPTB at less than 32 weeks of pregnancy, which was meaningful for the prognosis for newborns, due to the limited number of sPTB cases. Fourth, this study has not yet conducted a health economic evaluation. If we have the opportunity for large‐scale implementation, a health economic evaluation will be conducted in the future.

## AUTHOR CONTRIBUTIONS

SL contributed to conceptualization, data acquisition, data analysis, and manuscript writing and editing. YChen contributed to data acquisition, data analysis, and manuscript writing and editing. TJ, YChang, and WL contributed to investigation and manuscript editing. YP and XC contributed to conceptualization, project administration, data analysis, and manuscript writing and editing. All authors agree with the final version of the manuscript and its submission to the International Journal of Gynecology & Obstetrics.

## FUNDING INFORMATION

This work was supported by the National Natural Science Foundation of China (61802209), the Open Fund of Tianjin Central Hospital of Gynecology Obstetrics/Tianjin Key Laboratory of Human Development and Reproductive Regulation (2020XHY03), Tianjin Key Medical Discipline (Specialty) Construction Project (TJYXZDXK‐043A), Tianjin Municipal Education Commission Research Project (2023YXYZ07), National Key Research and Development Program of China (2021YFC2701500), and Tianjin Municipal Science and Technology Project (21JCZDJC00080).

## CONFLICT OF INTEREST STATEMENT

The authors have no conflicts of interest.

## Supporting information


Appendix S1.

## Data Availability

Research data are not shared.
