# Supplementary material for: Development of a spontaneous preterm birth predictive model using a panel of serum protein biomarkers for early pregnant women: A nested case–control study
Source: Int J Gynaecol Obstet. 2024 Aug 27;168(2):701–8. doi: 10.1002/ijgo.15876 (PMC11726131; doi:10.1002/ijgo.15876)
Supplement: Supplementary file 1 — Appendix S1. [file IJGO-168-701-s001.docx]

**Supplemental Methods**

**LC-MS/MS Analysis**

For model establishment, peptides were loaded onto a trap column (75 µm×20 mm, 3 µm C18,100 Å, 164535, Thermo Fisher Scientific) with a maximum pressure of 600 bar using mobile phase A (0.1% formic acid in H2O), separated on an analytical column (75 µm×150 mm, 3 µm C18,100 Å, 164568, Thermo Fisher Scientific) with a gradient of 5-55% mobile phase B (80% acetonitrile and 0.08% formic acid) at a flow rate of 300 nL/min for 130 min. A Q-Exactive HF-X mass spectrometer was programmed to acquire PRM combined with a full scan. Full scan acquisition was performed with a resolution of 60,000 at 200 m/z (3×10^6^ ions accumulated with a maximum injection time of 200 ms) to cover the scan range of 350-2000 m/z. PRM acquisition was performed using a resolution of 30,000 at 200 m/z, isolation windows of 0.4 m/z, target AGC values of 2×10^5^, and a maximum injection time of 120 ms. Fragmentation was performed with a normalized collision energy of 32 by HCD (high-energy collision dissociation).

For model validation, peptides were loaded and separated by the same HPLC system with a 120-min gradient. An Orbitrap Fusion Lumos Tribrid mass spectrometer was programmed in ms2 and ms3 PRM combined with a full scan. Full scan acquisition was performed with a resolution of 120,000 at 200 m/z (4×10^5^ ions accumulated with a maximum injection time of 50 ms) to cover the scan range of 350-1550 m/z. ms2 analysis was performed for the target m/z by using a resolution of 15,000 at 200 m/z, isolation windows of 0.7 m/z, target AGC values of 5×10^4^, and a maximum injection time of 22 ms. Collision-induced dissociation (CID) fragmentation was performed with a normalized collision energy of 35. Target ms3 analysis was performed using a resolution of 50,000 at 200 m/z, ms2 isolation windows of 3 m/z, target AGC values of 5×10^4^, and a maximum injection time of 86 ms. HCD fragmentation was performed with a normalized collision energy of 38.

The MS/MS data were searched against target peptide sequences using the SEQUEST search engine in Proteome Discoverer 2.3 software (PD2.3). The search criteria were as follows: full tryptic specificity was required; no missed cleavages were allowed; carbamidomethylation (C) and TMT6plex (K and N-terminal) were set as the fixed modifications; phosphorylation (S/T/Y) was set as the variable modification; precursor ion mass tolerances were set at 10 ppm for all MS acquired in an Orbitrap mass analyzer; and fragment ion mass tolerance was set at 20 mmu for all MS2 spectra acquired. Relative peptide quantification compared with the synthesized peptide standard was performed using PD2.3 based on the intensities of five reporter ions per peptide in the ms2 or ms3 spectra.

**Supplemental Table1. Biological process associated with candidate biomarkers**

| **Biological pathway** | **Candidate biomarker** | **Author, year, reference** |
| --- | --- | --- |
| Intrauterine infection/Inflammation | interleukin 1 alpha  Interleukin-10 | Ulla-Britt Wennerholm et al,1998[1]; K. Motomura et al,2020[2]  Goldenberg RL et al,2001[3] |
|  | Interleukin-15 | S. J. Fortunato et al,1998[4]; L. A. Gomez et al,2020[5] |
|  | Interleukin-17A  Interleukin-17F | L. A. Gomez et al,2020[5]; S. Park et al,2020[6];Yi Xu et al,2017[7]  L. A. Gomez et al,2020[5] |
|  | Interleukin-6 | Eliane Moura et al,2009[8]; Inglis SR et al,1994[9]; Thomakos N et al,2010[10] |
|  | Interleukin-1 beta | M. Schmid et al,2012[11]; Immaculate M et al,2016[12] |
|  | Interleukin-12 subunit beta | S. El-Shazly et al,2004[13] |
|  | Interleukin-2 | Curry AE et al,2009[14] |
|  | Interleukin-8 | Dowd J et al,2008[15]; Sakai M et al,2004[16]; Sakai M et al,2004[17] |
|  | Interleukin-5 | Tatiana Hountohotegbe et al,2020[18] |
|  | Interleukin-12 subunit alpha | S. El-Shazly et al,2004[13] |
|  | Interleukin-4 | V. S. Belousova et al,2019[19] |
|  | High mobility group protein B1 | E. Radnaa et al,2021[20] |
|  | Tumor necrosis factor | Eliane Moura et al,2009[8]; Inglis SR et al,1994[9]; Thomakos N et al,2010[10] |
|  | Interferon gamma | Eliane Moura et al,2009[8]; Curry AE et al,2009[14] |
|  | Alpha-2-HS-glycoprotein | Attila Molvarec et al, 2009[21] |
|  | Alpha-fetoprotein  Ceruloplasmin  Colony-stimulating factor-2  C-X-C motif chemokine ligand 10  transthyretin | Jilin Hu, et al,2019[22],  A. Seval Ozgu-Erdinc et al,2014[23]  Rachel G. Sinkey et al,2020[24]  Stefania Ronzoni et al,2018[25]  F. J. Rosales et al, 1996[26] ; A. Myron Johnson et al,2007[27] |
| Placental protein/ hormona –related  biomarker | Corticoliberin | Wei Perng et al,2020[28] |
|  | Sex hormone-binding globulin | George R. Saade et al,2016[29] |
|  | Progesterone-induced-blocking factor 1  Insulin-like growth factor-binding protein 4  Insulin-like growth factor-binding protein1  phI-Insulin-like growth factor-binding protein 1  Pregnancy-specific beta-1-glycoprotein 3  Pregnancy-specific beta-1-glycoprotein 4  Pregnancy associated plasma protein –A  chorionic gonadotropin subunit beta 3 | B. Huang et al,2017[30];  George R. Saade et al,2016[29]  R. Devliegeret al,2009[31]; D. Balic et al,2008[32]  D. Paternoster et al,2009[33]; V. Wiwanitkit,2010[34]; O. Altinkaya et al,2009[35]  J. Warren2018[36]  J. Warren2018[36]  Marja Kaijomaa et al,[37]; Alice E. Hughes et al, 2019[38]  Ida Kirkegaard et al,2010[39]; Gayathri Rengaraj et al,2007[40] |
| Immunity | C-X-C motif chemokine 9 | S. Ronzoni et al,2019[25] |
|  | C-X-C chemokine receptor type 5  C-X-C motif chemokine 13 | C. Silwedel et al,2019[41]  Y. Luo et al,2020[42] |
| Matrix remodeling | matrix metalloproteinase 8  matrix metalloproteinase 9 | Yoon BH et al,2001[43]  H. J. Kim et al,2020[44]; J. W. Park et al,2019[45] |
|  | Fibulin-1  vitronectin | Satoko Ito et al,2020[46]  Kimie Date et al, 2019[47]; Teresa Cobo et al, 2018[48] |
| Angiogenesis disorders | Vascular endothelial growth factor receptor 1 | Sean Lim et al,2021[49]; C. Villalain et al,2020[50] |
|  | Endoglin | T. Chaiworapongsa et al,2009[51]; |
|  | Dickkopf-related protein 1 | SHENG-JUN JIANG et al,2015[52]; |
|  | Interferon alpha-1-13  Alpha-fetoprotein  placental growth factor | Danieli Andrade et al,2015[53]  OLIN D. LIANG et al,2004[54]  Mahsa Matin et al,2020[55]; A. Leanos-Miranda et al,2020[56]; John R. Barton et al,[57] |

**Supplemental Table 2. Peptide list of 44 PTB potential biomarkers**

| **NO** | **Uniprot Accession** | **Gene Name** | **Peptide** |
| --- | --- | --- | --- |
| 1 | P02771 | AFP | GYQELLEK |
| 2 | Q13219 | PAPPA | ALYFSGR |
| 3 | P17948 | FLT1 | GFIISNATYK |
| 4 | P05231 | IL6 | YILDGISALR |
| 5 | P01584 | IL1B | ISDHHYSK |
| 6 | P51911 | CNN1 | LQPGSVK |
| 7 | P06850 | CRH | MGEEYFLR |
| 8 | P60568 | IL2 | WITFCQSIISTLT |
| 9 | P10145 | CXCL8 | VIESGPHCANTEIIVK |
| 10 | P22894 | MMP8 | TVQDYLEK |
| 11 | P14780 | MMP9 | QLAEEYLYR |
| 12 | Q8WXW3 | PIBF1 | ELQLSTESK |
| 13 | P0DN86 | CGB3 | APPPSLPSPSR |
| 14 | P02765 | AHSG | EATEAAK |
| 15 | P00450 | CP | DNEDFQESNR |
| 16 | P23142 | FBLN1 | TGYYFDGISR |
| 17 | P49763 | PGF | CECRPLR |
| 18 | Q16557 | PSG3 | LFIPQITTK |
| 19 | Q00888 | PSG4 | TLFIFGVTK |
| 20 | P17813 | ENG | GEVTYTTSQVSK |
| 21 | P02766 | TTR | VLDAVR |
| 22 | P04004 | VTN | NGSLFAFR |
| 23 | P02778 | CXCL10 | CLNPESK |
| 24 | P29460 | IL12B | TLTIQVK |
| 25 | P01579 | IFNG | DDQSIQK |
| 26 | O43927 | CXCL13 | SIVCVDPQAEWIQR |
| 27 | Q07325 | CXCL9 | IEIIATLK |
| 28 | P32302 | CXCR5 | TVIALHK |
| 29 | O94907 | DKK1 | GQEGSVCLR |
| 30 | P01562 | IFNA1 | ITLYLTEK |
| 31 | P29459 | IL12A | AVSNMLQK |
| 32 | P05112 | IL4 | TLNSLTEQK |
| 33 | P05113 | IL5 | ETLALLSTHR |
| 34 | P09429 | HMGB1 | GEHPGLSIGDVAK |
| 35 | P01583 | IL1A | FDMGAYK |
| 36 | P01375 | TNF | VNLLSAIK |
| 37 | P04141 | CSF2 | LLNLSR |
| 38 | P22301 | IL10 | ESLLEDFK |
| 39 | P40933 | IL15 | ECEELEEK |
| 40 | Q16552 | IL17A | YPSVIWEAK |
| 41 | Q96PD4 | IL17F | LDIGIINENQR |
| 42 | P08833 | IGFBP1 | AQETSGEEISK |
| 43 | P08833 | IGFBP1 | AQETS(p)GEEISK |
| 44 | P04278 | SHBG | QAEISASAPTSLR |
| 45 | P22692 | IGFBP4 | QCHPALDGQR |
| 46 | P04278 | SHBG | VVLSQGSK |
| 47 | P04278 | SHBG | LDVDQALNR |
| 48 | P22692 | IGFBP4 | LPGGLEPK |

AQETS(p)GEEISK stand for that the 5^th^ S is phosphorylated.

**Supplemental Table 3.** Clinical characteristics of the population of scalability set

| Preterm (n=27) Term (n=45) | | | | |
| --- | --- | --- | --- | --- |
| Age  Prepregnancy BMI  <18.5  18.5–24.9  ≥25  Height  Educational level  <College  =College  >College  Cigarette smoking  Multigravida  Preterm delivery history  Term delivery history  Primigravida  Assisted reproduction  Gestational days at serum collection  Bleeding during pregnancy  before 12 wk |  | 32.846 ±0.780  2 (7.4%)  19 (70.4%)  6 (22.2%)  162.250±1.316  2 (22.2%)  4 (14.8%)  20 (74.17%)  3（11.1%）  1 (0.0%)  11  2 (7.4%)  9（33.3%）  16 (59.3%)  4 (14.8%)  191.33±6.25  6 (22.2% | 32.289±0.672  5 (11.1%)  30 (66. 7%)  10(22.2%)  163.806±0.650  2 (22.2%)  5(11.1%)  35 (77.8%)  5（11.1%）  1 (0.0%)  14  2 (4.4%)  12（26.6%）  31 (68.9%)  2 (4.4%)  198.87±4.26  9 (20.2%) |  |

**Supplemental Table 4**. Model performances in terms of classification accuracy, sensitivity, specificity, precision, and F-measure on scalability validation set (20-32 GW).

| **Models** | Acc | Sen | Spe | Precision | F-measure |
| --- | --- | --- | --- | --- | --- |
| **LR** | 0.847 | 0.720 | 0.915 | 0.818 | 0.766 |
| **SVM** | 0.764 | 0.760 | 0.766 | 0.633 | 0.691 |
| **RF** | 0.792 | 0.720 | 0.830 | 0.692 | 0.706 |

^*Acc: Accuracy, Sen: Sensitivity, Spe: Specificity, LR: Logistic regression, SVM: Support vector machine, RF: Random forest^


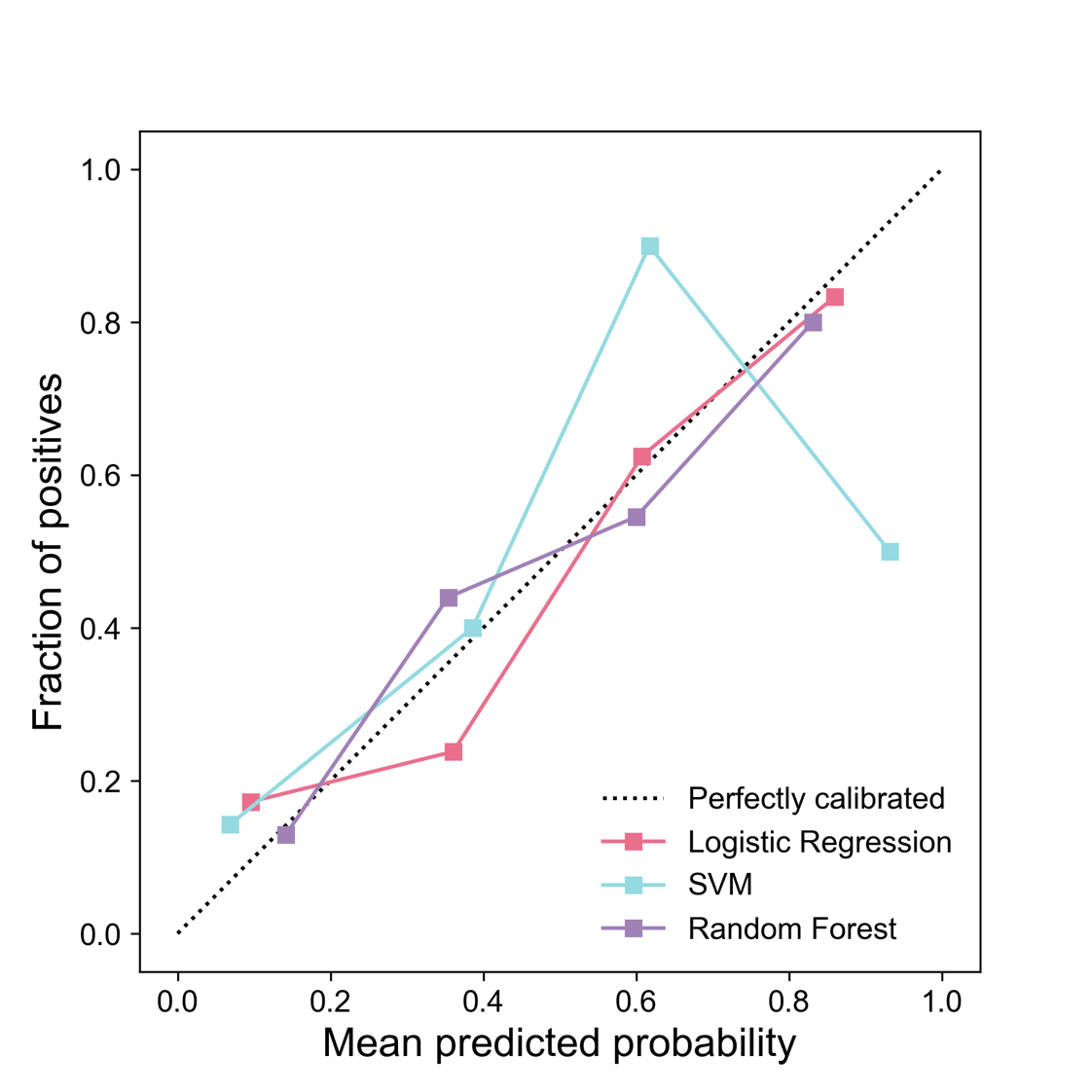


**Supplemental Figure 1**. Calibration plots of three models on scalability evaluation set (20-32 GW).

**Reference**

1. Wennerholm, U. B.; Holm, B.; Mattsby-Baltzer, I.; Nielsen, T.; Platz-Christensen, J. J.; Sundell, G.; Hagberg, H., Interleukin-1alpha, interleukin-6 and interleukin-8 in cervico/vaginal secretion for screening of preterm birth in twin gestation. *Acta Obstet Gynecol Scand* **1998,** 77, (5), 508-14.

2. Motomura, K.; Romero, R.; Garcia-Flores, V.; Leng, Y.; Xu, Y.; Galaz, J.; Slutsky, R.; Levenson, D.; Gomez-Lopez, N., The alarmin interleukin-1alpha causes preterm birth through the NLRP3 inflammasome. *Mol Hum Reprod* **2020,** 26, (9), 712-726.

3. Goldenberg, R. L.; Iams, J. D.; Mercer, B. M.; Meis, P. J.; Moawad, A.; Das, A.; Miodovnik, M.; Vandorsten, P. J.; Caritis, S. N.; Thurnau, G.; Dombrowski, M. P.; Maternal-Fetal Medicine Units, N., The Preterm Prediction Study: toward a multiple-marker test for spontaneous preterm birth. *Am J Obstet Gynecol* **2001,** 185, (3), 643-51.

4. Fortunato, S. J.; Menon, R.; Lombardi, S. J., IL-15, a novel cytokine produced by human fetal membranes, is elevated in preterm labor. *Am J Reprod Immunol* **1998,** 39, (1), 16-23.

5. Gomez, L. A.; De Avila, J.; Castillo, D. M.; Montenegro, D. A.; Trujillo, T. G.; Suarez, L. J.; Lafaurie, G. I., Porphyromonas gingivalis Placental Atopobiosis and Inflammatory Responses in Women With Adverse Pregnancy Outcomes. *Front Microbiol* **2020,** 11, 591626.

6. Park, S.; You, Y. A.; Yun, H.; Choi, S. J.; Hwang, H. S.; Choi, S. K.; Lee, S. M.; Kim, Y. J., Cervicovaginal fluid cytokines as predictive markers of preterm birth in symptomatic women. *Obstet Gynecol Sci* **2020,** 63, (4), 455-463.

7. Xu, Y.; Romero, R.; Miller, D.; Silva, P.; Panaitescu, B.; Theis, K. R.; Arif, A.; Hassan, S. S.; Gomez-Lopez, N., Innate lymphoid cells at the human maternal-fetal interface in spontaneous preterm labor. *Am J Reprod Immunol* **2018,** 79, (6), e12820.

8. Moura, E.; Mattar, R.; de Souza, E.; Torloni, M. R.; Goncalves-Primo, A.; Daher, S., Inflammatory cytokine gene polymorphisms and spontaneous preterm birth. *J Reprod Immunol* **2009,** 80, (1-2), 115-21.

9. Inglis, S. R.; Jeremias, J.; Kuno, K.; Lescale, K.; Peeper, Q.; Chervenak, F. A.; Witkin, S. S., Detection of tumor necrosis factor-alpha, interleukin-6, and fetal fibronectin in the lower genital tract during pregnancy: relation to outcome. *Am J Obstet Gynecol* **1994,** 171, (1), 5-10.

10. Thomakos, N.; Daskalakis, G.; Papapanagiotou, A.; Papantoniou, N.; Mesogitis, S.; Antsaklis, A., Amniotic fluid interleukin-6 and tumor necrosis factor-alpha at mid-trimester genetic amniocentesis: relationship to intra-amniotic microbial invasion and preterm delivery. *Eur J Obstet Gynecol Reprod Biol* **2010,** 148, (2), 147-51.

11. Schmid, M.; Haslinger, P.; Stary, S.; Leipold, H.; Egarter, C.; Grimm, C., Interleukin-1 beta gene polymorphisms and preterm birth. *Eur J Obstet Gynecol Reprod Biol* **2012,** 165, (1), 33-6.

12. Langmia, I. M.; Apalasamy, Y. D.; Omar, S. Z.; Mohamed, Z., Impact of IL1B gene polymorphisms and interleukin 1B levels on susceptibility to spontaneous preterm birth. *Pharmacogenet Genomics* **2016,** 26, (11), 505-509.

13. El-Shazly, S.; Makhseed, M.; Azizieh, F.; Raghupathy, R., Increased expression of pro-inflammatory cytokines in placentas of women undergoing spontaneous preterm delivery or premature rupture of membranes. *Am J Reprod Immunol* **2004,** 52, (1), 45-52.

14. Curry, A. E.; Thorsen, P.; Drews, C.; Schendel, D.; Skogstrand, K.; Flanders, W. D.; Hougaard, D.; Olsen, J.; Vogel, I., First-trimester maternal plasma cytokine levels, pre-pregnancy body mass index, and spontaneous preterm delivery. *Acta Obstet Gynecol Scand* **2009,** 88, (3), 332-42.

15. Dowd, J.; Laham, N.; Rice, G.; Brennecke, S.; Permezel, M., Elevated interleukin-8 concentrations in cervical secretions are associated with preterm labour. *Gynecol Obstet Invest* **2001,** 51, (3), 165-8.

16. Sakai, M.; Sasaki, Y.; Yoneda, S.; Kasahara, T.; Arai, T.; Okada, M.; Hosokawa, H.; Kato, K.; Soeda, Y.; Saito, S., Elevated interleukin-8 in cervical mucus as an indicator for treatment to prevent premature birth and preterm, pre-labor rupture of membranes: a prospective study. *Am J Reprod Immunol* **2004,** 51, (3), 220-5.

17. Sakai, M.; Ishiyama, A.; Tabata, M.; Sasaki, Y.; Yoneda, S.; Shiozaki, A.; Saito, S., Relationship between cervical mucus interleukin-8 concentrations and vaginal bacteria in pregnancy. *Am J Reprod Immunol* **2004,** 52, (2), 106-12.

18. Hountohotegbe, T.; Gbedande, K.; Agbota, G.; Ibitokou, S.; Massougbodji, A.; Deloron, P.; Fievet, N.; Luty, A. J. F., Circulating Cytokines Associated with Poor Pregnancy Outcomes in Beninese Exposed to Infection with Plasmodium falciparum. *Infect Immun* **2020,** 88, (8).

19. Belousova, V. S.; Svitich, O. A.; Timokhina, E. V.; Strizhakov, A. N.; Bogomazova, I. M., Polymorphism of the IL-1beta, TNF, IL-1RA and IL-4 Cytokine Genes Significantly Increases the Risk of Preterm Birth. *Biochemistry (Mosc)* **2019,** 84, (9), 1040-1046.

20. Radnaa, E.; Richardson, L. S.; Sheller-Miller, S.; Baljinnyam, T.; de Castro Silva, M.; Kumar Kammala, A.; Urrabaz-Garza, R.; Kechichian, T.; Kim, S.; Han, A.; Menon, R., Extracellular vesicle mediated feto-maternal HMGB1 signaling induces preterm birth. *Lab Chip* **2021,** 21, (10), 1956-1973.

21. Molvarec, A.; Kalabay, L.; Derzsy, Z.; Szarka, A.; Halmos, A.; Stenczer, B.; Arnaud, P.; Karadi, I.; Prohaszka, Z.; Rigo, J., Jr., Preeclampsia is associated with decreased serum alpha(2)-HS glycoprotein (fetuin-A) concentration. *Hypertens Res* **2009,** 32, (8), 665-9.

22. Hu, J.; Zhang, J.; Chan, Y.; Zhu, B., A rat model of placental inflammation explains the unexplained elevated maternal serum alpha-fetoprotein associated with adverse pregnancy outcomes. *J Obstet Gynaecol Res* **2019,** 45, (10), 1980-1988.

23. Ozgu-Erdinc, A. S.; Cavkaytar, S.; Aktulay, A.; Buyukkagnici, U.; Erkaya, S.; Danisman, N., Mid-trimester maternal serum and amniotic fluid biomarkers for the prediction of preterm delivery and intrauterine growth retardation. *J Obstet Gynaecol Res* **2014,** 40, (6), 1540-6.

24. Sinkey, R. G.; Guzeloglu-Kayisli, O.; Arlier, S.; Guo, X.; Semerci, N.; Moore, R.; Ozmen, A.; Larsen, K.; Nwabuobi, C.; Kumar, D.; Moore, J. J.; Buckwalder, L. F.; Schatz, F.; Kayisli, U. A.; Lockwood, C. J., Thrombin-Induced Decidual Colony-Stimulating Factor-2 Promotes Abruption-Related Preterm Birth by Weakening Fetal Membranes. *Am J Pathol* **2020,** 190, (2), 388-399.

25. Ronzoni, S.; Steckle, V.; D'Souza, R.; Murphy, K. E.; Lye, S.; Shynlova, O., Cytokine Changes in Maternal Peripheral Blood Correlate With Time-to-Delivery in Pregnancies Complicated by Premature Prelabor Rupture of the Membranes. *Reprod Sci* **2019,** 26, (9), 1266-1276.

26. Rosales, F. J.; Ritter, S. J.; Zolfaghari, R.; Smith, J. E.; Ross, A. C., Effects of acute inflammation on plasma retinol, retinol-binding protein, and its mRNA in the liver and kidneys of vitamin A-sufficient rats. *J Lipid Res* **1996,** 37, (5), 962-71.

27. Myron Johnson, A.; Merlini, G.; Sheldon, J.; Ichihara, K.; Scientific Division Committee on Plasma Proteins, I. F. o. C. C.; Laboratory, M., Clinical indications for plasma protein assays: transthyretin (prealbumin) in inflammation and malnutrition. *Clin Chem Lab Med* **2007,** 45, (3), 419-26.

28. Perng, W.; Holzman, C.; Talge, N. M.; Senagore, P. K., Placental pathology, corticotropin-releasing hormone, timing of parturition, and fetal growth in the pregnancy outcomes and community health study. *J Matern Fetal Neonatal Med* **2020,** 33, (7), 1225-1232.

29. Saade, G. R.; Boggess, K. A.; Sullivan, S. A.; Markenson, G. R.; Iams, J. D.; Coonrod, D. V.; Pereira, L. M.; Esplin, M. S.; Cousins, L. M.; Lam, G. K.; Hoffman, M. K.; Severinsen, R. D.; Pugmire, T.; Flick, J. S.; Fox, A. C.; Lueth, A. J.; Rust, S. R.; Mazzola, E.; Hsu, C.; Dufford, M. T.; Bradford, C. L.; Ichetovkin, I. E.; Fleischer, T. C.; Polpitiya, A. D.; Critchfield, G. C.; Kearney, P. E.; Boniface, J. J.; Hickok, D. E., Development and validation of a spontaneous preterm delivery predictor in asymptomatic women. *Am J Obstet Gynecol* **2016,** 214, (5), 633 e1-633 e24.

30. Huang, B.; Faucette, A. N.; Pawlitz, M. D.; Pei, B.; Goyert, J. W.; Zhou, J. Z.; El-Hage, N. G.; Deng, J.; Lin, J.; Yao, F.; Dewar, R. S., 3rd; Jassal, J. S.; Sandberg, M. L.; Dai, J.; Cols, M.; Shen, C.; Polin, L. A.; Nichols, R. A.; Jones, T. B.; Bluth, M. H.; Puder, K. S.; Gonik, B.; Nayak, N. R.; Puscheck, E.; Wei, W. Z.; Cerutti, A.; Colonna, M.; Chen, K., Interleukin-33-induced expression of PIBF1 by decidual B cells protects against preterm labor. *Nat Med* **2017,** 23, (1), 128-135.

31. Devlieger, R.; Verhaeghe, J.; Coopmans, W.; Deprest, J. A., IGFBP-1 levels in cervicovaginal secretions before and after amniocentesis. *Gynecol Obstet Invest* **2009,** 67, (1), 9-13.

32. Balic, D.; Latifagic, A.; Hudic, I., Insulin-like growth factor-binding protein-1 (IGFBP-1) in cervical secretions as a predictor of preterm delivery. *J Matern Fetal Neonatal Med* **2008,** 21, (5), 297-300.

33. Paternoster, D.; Riboni, F.; Vitulo, A.; Plebani, M.; Dell'Avanzo, M.; Battagliarin, G.; Surico, N.; Nicolini, U., Phosphorylated insulin-like growth factor binding protein-1 in cervical secretions and sonographic cervical length in the prediction of spontaneous preterm delivery. *Ultrasound Obstet Gynecol* **2009,** 34, (4), 437-40.

34. Wiwanitkit, V., Diagnostic property of cervical phosphorylated insulin-like growth factor binding protein-1 in the prediction of preterm labor in symptomatic patients. *Arch Gynecol Obstet* **2010,** 281, (1), 175-6.

35. Altinkaya, O.; Gungor, T.; Ozat, M.; Danisman, N.; Mollamahmutoglu, L., Cervical phosphorylated insulin-like growth factor binding protein-1 in prediction of preterm delivery. *Arch Gynecol Obstet* **2009,** 279, (3), 279-83.

36. Warren, J.; Im, M.; Ballesteros, A.; Ha, C.; Moore, T.; Lambert, F.; Lucas, S.; Hinz, B.; Dveksler, G., Activation of latent transforming growth factor-beta1, a conserved function for pregnancy-specific beta 1-glycoproteins. *Mol Hum Reprod* **2018,** 24, (12), 602-612.

37. Kaijomaa, M.; Ulander, V. M.; Hamalainen, E.; Alfthan, H.; Markkanen, H.; Heinonen, S.; Stefanovic, V., The risk of adverse pregnancy outcome among pregnancies with extremely low maternal PAPP-A. *Prenat Diagn* **2016,** 36, (12), 1115-1120.

38. Hughes, A. E.; Sovio, U.; Gaccioli, F.; Cook, E.; Charnock-Jones, D. S.; Smith, G. C. S., The association between first trimester AFP to PAPP-A ratio and placentally-related adverse pregnancy outcome. *Placenta* **2019,** 81, 25-31.

39. Kirkegaard, I.; Uldbjerg, N.; Petersen, O. B.; Torring, N.; Henriksen, T. B., PAPP-A, free beta-hCG, and early fetal growth identify two pathways leading to preterm delivery. *Prenat Diagn* **2010,** 30, (10), 956-63.

40. Rengaraj, G.; Guleria, K.; Suneja, A.; Gambhir, J. K., Human chorionic gonadotropin in cervicovaginal secretions as a predictor of preterm birth. *Gynecol Obstet Invest* **2009,** 67, (3), 202-7.

41. Silwedel, C.; Speer, C. P.; Haarmann, A.; Fehrholz, M.; Claus, H.; Schlegel, N.; Glaser, K., Ureaplasma Species Modulate Cytokine and Chemokine Responses in Human Brain Microvascular Endothelial Cells. *Int J Mol Sci* **2019,** 20, (14).

42. Luo, Y.; Luo, F.; Zhang, K.; Wang, S.; Zhang, H.; Yang, X.; Shang, W.; Wang, J.; Wang, Z.; Pang, X.; Feng, Y.; Liu, L.; Xie, H.; Feng, G.; Li, J., Elevated Circulating IL-10 Producing Breg, but Not Regulatory B Cell Levels, Restrain Antibody-Mediated Rejection After Kidney Transplantation. *Front Immunol* **2020,** 11, 627496.

43. Yoon, B. H.; Oh, S. Y.; Romero, R.; Shim, S. S.; Han, S. Y.; Park, J. S.; Jun, J. K., An elevated amniotic fluid matrix metalloproteinase-8 level at the time of mid-trimester genetic amniocentesis is a risk factor for spontaneous preterm delivery. *Am J Obstet Gynecol* **2001,** 185, (5), 1162-7.

44. Kim, H. J.; Park, K. H.; Kim, Y. M.; Joo, E.; Ahn, K.; Shin, S., A protein microarray analysis of amniotic fluid proteins for the prediction of spontaneous preterm delivery in women with preterm premature rupture of membranes at 23 to 30 weeks of gestation. *PLoS One* **2020,** 15, (12), e0244720.

45. Park, J. W.; Park, K. H.; Lee, J. E.; Kim, Y. M.; Lee, S. J.; Cheon, D. H., Antibody Microarray Analysis of Plasma Proteins for the Prediction of Histologic Chorioamnionitis in Women With Preterm Premature Rupture of Membranes. *Reprod Sci* **2019,** 26, (11), 1476-1484.

46. Ito, S.; Yokoyama, U.; Nakakoji, T.; Cooley, M. A.; Sasaki, T.; Hatano, S.; Kato, Y.; Saito, J.; Nicho, N.; Iwasaki, S.; Umemura, M.; Fujita, T.; Masuda, M.; Asou, T.; Ishikawa, Y., Fibulin-1 Integrates Subendothelial Extracellular Matrices and Contributes to Anatomical Closure of the Ductus Arteriosus. *Arterioscler Thromb Vasc Biol* **2020,** 40, (9), 2212-2226.

47. Date, K.; Suzuki, R.; Oda-Tamai, S.; Ogawa, H., Vitronectins produced by human cirrhotic liver and CCl4-treated rats differ in their glycosylation pattern and tissue remodeling activity. *FEBS Open Bio* **2019,** 9, (4), 755-768.

48. Cobo, T.; Palacio, M.; Grande, M.; Sanchez-Garcia, A. B.; Estanyol, J. M.; Lopez, M.; Bosch, J.; Marti, C.; Gratacos, E., Cervical Alpha-Actinin-4 Is Upregulated in Women with Threatened Preterm Labor and Microbial Invasion of the Amniotic Cavity. *Fetal Diagn Ther* **2018,** 44, (1), 36-43.

49. Lim, S.; Li, W.; Kemper, J.; Nguyen, A.; Mol, B. W.; Reddy, M., Biomarkers and the Prediction of Adverse Outcomes in Preeclampsia: A Systematic Review and Meta-analysis. *Obstet Gynecol* **2021,** 137, (1), 72-81.

50. Villalain, C.; Herraiz, I.; Cantero, B.; Quezada, S.; Lopez, A.; Simon, E.; Galindo, A., Angiogenesis biomarkers for the prediction of severe adverse outcomes in late-preterm preeclampsia. *Pregnancy Hypertens* **2020,** 19, 74-80.

51. Chaiworapongsa, T.; Romero, R.; Tarca, A.; Kusanovic, J. P.; Mittal, P.; Kim, S. K.; Gotsch, F.; Erez, O.; Vaisbuch, E.; Mazaki-Tovi, S.; Pacora, P.; Ogge, G.; Dong, Z.; Kim, C. J.; Yeo, L.; Hassan, S. S., A subset of patients destined to develop spontaneous preterm labor has an abnormal angiogenic/anti-angiogenic profile in maternal plasma: evidence in support of pathophysiologic heterogeneity of preterm labor derived from a longitudinal study. *J Matern Fetal Neonatal Med* **2009,** 22, (12), 1122-39.

52. Jiang, S. J.; Li, W.; Li, Y. J.; Fang, W.; Long, X., Dickkopfrelated protein 1 induces angiogenesis by upregulating vascular endothelial growth factor in the synovial fibroblasts of patients with temporomandibular joint disorders. *Mol Med Rep* **2015,** 12, (4), 4959-66.

53. Andrade, D.; Kim, M.; Blanco, L. P.; Karumanchi, S. A.; Koo, G. C.; Redecha, P.; Kirou, K.; Alvarez, A. M.; Mulla, M. J.; Crow, M. K.; Abrahams, V. M.; Kaplan, M. J.; Salmon, J. E., Interferon-alpha and angiogenic dysregulation in pregnant lupus patients who develop preeclampsia. *Arthritis Rheumatol* **2015,** 67, (4), 977-87.

54. Liang, O. D.; Korff, T.; Eckhardt, J.; Rifaat, J.; Baal, N.; Herr, F.; Preissner, K. T.; Zygmunt, M., Oncodevelopmental alpha-fetoprotein acts as a selective proangiogenic factor on endothelial cell from the fetomaternal unit. *J Clin Endocrinol Metab* **2004,** 89, (3), 1415-22.

55. Matin, M.; Morgelin, M.; Stetefeld, J.; Schermer, B.; Brinkkoetter, P. T.; Benzing, T.; Koch, M.; Hagmann, H., Affinity-Enhanced Multimeric VEGF (Vascular Endothelial Growth Factor) and PlGF (Placental Growth Factor) Variants for Specific Adsorption of sFlt-1 to Restore Angiogenic Balance in Preeclampsia. *Hypertension* **2020,** 76, (4), 1176-1184.

56. Leanos-Miranda, A.; Graciela Nolasco-Leanos, A.; Ismael Carrillo-Juarez, R.; Jose Molina-Perez, C.; Janet Sillas-Pardo, L.; Manuel Jimenez-Trejo, L.; Isordia-Salas, I.; Leticia Ramirez-Valenzuela, K., Usefulness of the sFlt-1/PlGF (Soluble fms-Like Tyrosine Kinase-1/Placental Growth Factor) Ratio in Diagnosis or Misdiagnosis in Women With Clinical Diagnosis of Preeclampsia. *Hypertension* **2020,** 76, (3), 892-900.

57. Barton, J. R.; Woelkers, D. A.; Newman, R. B.; Combs, C. A.; How, H. Y.; Boggess, K. A.; Martin, J. N., Jr.; Kupfer, K.; Sibai, B. M.; Trial, P., Placental growth factor predicts time to delivery in women with signs or symptoms of early preterm preeclampsia: a prospective multicenter study. *Am J Obstet Gynecol* **2020,** 222, (3), 259 e1-259 e11.
